# Longitudinal analysis of subtype C envelope tropism for memory CD4^+^ T cell subsets over the first 3 years of untreated HIV-1 infection

**DOI:** 10.1186/s12977-020-00532-2

**Published:** 2020-08-06

**Authors:** Matthew J. Gartner, Paul R. Gorry, Carolin Tumpach, Jingling Zhou, Ashanti Dantanarayana, J. Judy Chang, Thomas A. Angelovich, Paula Ellenberg, Annemarie E. Laumaea, Molati Nonyane, Penny L. Moore, Sharon R. Lewin, Melissa J. Churchill, Jacqueline K. Flynn, Michael Roche

**Affiliations:** 1 School of Health and Biomedical Sciences, RMIT University, Bundoora, Melbourne, VIC Australia; 2 The Peter Doherty Institute for Infection and Immunity, University of Melbourne and Royal Melbourne Hospital, Melbourne, VIC Australia; 3 Life Sciences, Burnet Institute, Melbourne, VIC Australia; 4 Département de Microbiologie, Infectiologie et Immunologie, Université de Montréal, Montreal, QC Canada; 5 Centre for HIV and STIs, National Institute for Communicable Diseases (NICD) of the National Health Laboratory Service (NHLS), Johannesburg, South Africa; 6 Faculty of Health Sciences, University of the Witwatersrand, Johannesburg, South Africa; 7 Centre for the AIDS Programme of Research in South Africa (CAPRISA), University of KwaZulu-Natal, Durban, South Africa; 8 Department of Infectious Diseases, Monash University and Alfred Hospital, Melbourne, Australia; 9 School of Clinical Sciences, Monash University, Melbourne, VIC Australia

**Keywords:** Cellular tropism, CD4^+^ T cells, Subtype C HIV-1, Coreceptor usage, Envelope

## Abstract

**Background:**

HIV-1 infects a wide range of CD4^+^ T cells with different phenotypic properties and differing expression levels of entry coreceptors. We sought to determine the viral tropism of subtype C (C-HIV) Envelope (Env) clones for different CD4^+^ T cell subsets and whether tropism changes during acute to chronic disease progression. HIV-1 *envs* were amplified from the plasma of five C-HIV infected women from three untreated time points; less than 2 months, 1-year and 3-years post-infection. Pseudoviruses were generated from Env clones, phenotyped for coreceptor usage and CD4^+^ T cell subset tropism was measured by flow cytometry.

**Results:**

A total of 50 C-HIV *envs* were cloned and screened for functionality in pseudovirus infection assays. Phylogenetic and variable region characteristic analysis demonstrated evolution in *envs* between time points. We found 45 pseudoviruses were functional and all used CCR5 to mediate entry into NP2/CD4/CCR5 cells. In vitro infection assays showed transitional memory (TM) and effector memory (EM) CD4^+^ T cells were more frequently infected (median: 46% and 25% of total infected CD4^+^ T cells respectively) than naïve, stem cell memory, central memory and terminally differentiated cells. This was not due to these subsets contributing a higher proportion of the CD4^+^ T cell pool, rather these subsets were more susceptible to infection (median: 5.38% EM and 2.15% TM cells infected), consistent with heightened CCR5 expression on EM and TM cells. No inter- or intra-participant changes in CD4^+^ T cell subset tropism were observed across the three-time points.

**Conclusions:**

CD4^+^ T cell subsets that express more CCR5 were more susceptible to infection with C-HIV Envs, suggesting that these may be the major cellular targets during the first 3 years of infection. Moreover, we found that viral tropism for different CD4^+^ T cell subsets in vitro did not change between Envs cloned from acute to chronic disease stages. Finally, central memory, naïve and stem cell memory CD4^+^ T cell subsets were susceptible to infection, albeit inefficiently by Envs from all time-points, suggesting that direct infection of these cells may help establish the latent reservoir early in infection.

## Background

HIV-1 infects a broad range of CD4^+^ T cells. Activated CD4^+^ T cells are highly susceptible to infection, and most die within 2–3 days of infection [1–3]. In contrast, long-lived resting memory CD4^+^ T cells are less frequently infected [4, 5] and are primary targets for latent infection [6, 7]. Latent infection allows HIV-1 to persist during suppressive antiretroviral therapy (ART) [8]. Furthermore, latently infected cells serve as the source of viral recrudescence when ART is interrupted. As such, the latent reservoir represents a significant barrier to achieving HIV-1 cure using current therapeutic strategies.

HIV-1 enters host cells through engagement of the surface exposed region of gp120 in the Envelope (Env) trimer with the CD4 receptor on immune cells [9]. This gp120-CD4 interaction leads to conformational changes within Env that expose the V3 loop for binding with a coreceptor [10], either CCR5 and/or CXCR4 [11–13]. Interaction between the Env and coreceptor leads to fusion between the viral and cellular membranes [14]. Given that viruses can use CCR5 and/or CXCR4 to mediate entry, they are categorised by their ability to use one or both coreceptors (CCR5-using, CXCR4-using, or dual-tropic). Viruses isolated from early stages of infection are almost exclusively CCR5-using [15–20]. However, in 50% of untreated subtype B infections, CXCR4-using viruses can emerge either exclusively or with CCR5-using viruses [21]. In contrast, coreceptor switching to CXCR4 occurs less frequently in subtype C (C-HIV) infections [22, 23]. Moreover, several studies have shown CCR5-using Envs from chronic disease stages of subtype B and C engage CCR5 differentially compared to Envs from acute infection [15, 16, 24]. In some cases this differential engagement may lead to improved infectivity of cells expressing lower levels of CCR5 during chronic infection [20].

Following antigen stimulation, naive CD4^+^ T cells proliferate and differentiate into several T helper subsets with distinct effector functions [25]. Most of these effector cells die following the elimination of antigen, although a small proportion of these cells survive to become long-lived memory CD4^+^ T cells [26]. Memory CD4^+^ T cell subsets include stem cell memory (TSCM), central memory (CM), transitional memory (TM), effector memory (EM), and terminally differentiated (TD) [27–29]. These subsets display variable susceptibilities to HIV-1 infection [18, 19], which can be influenced by differential cellular activation status [30], tissue localisation [31] and coreceptor expression [32, 33]. Although HIV-1 DNA can be found in all of these subsets during suppressive ART, less differentiated subsets (i.e. naïve, TSCM and CM) have been implicated as highly stable reservoirs [34–37]. These cells express low levels of CCR5 in comparison to more differentiated memory T cell subsets [32, 33, 38, 39], and thus it is unclear whether these cells are directly infected or if infection requires some other stimulus to increase CCR5 expression [40]. Additionally, it is unknown whether viruses change their preference for different memory CD4^+^ T cell subsets from early to chronic disease stages during untreated infection.

In this study, we assessed the viral tropism of longitudinal C-HIV Envs for memory CD4^+^ T cell subsets in vitro, and whether subset tropism changes over time. We hypothesised that Envs derived from participants during acute infection would show a preference for infecting more differentiated subsets (TM and EM) given their high CCR5 expression [32, 33] and that as disease progressed, Envs would evolve to use CCR5 more efficiently to infect less differentiated CD4^+^ T cells (i.e. naïve, TSCM and CM). *Env* sequences were generated using single genome amplification (SGA) from the plasma of five untreated South African women living with C-HIV enrolled in the CAPRISA 002 Acute infection cohort from three-time points: less than 2 months (acute infection), 1-year and 3 years’ post-infection. *Envs* were pseudotyped onto the same reporter virus backbone to determine functionality, coreceptor usage and memory CD4^+^ T cell tropism. We found that all viruses were CCR5-using with only three viruses from one participant also showing weak CXCR4-usage. Infection assays in CD4^+^ T cells revealed that TM and EM cells were most frequently infected by all pseudoviruses (46% and 25% of total infected cells respectively) compared to other subsets. We saw no change in memory CD4^+^ T cell subset tropism during acute to chronic disease progression. Our data suggest that more differentiated memory CD4^+^ T cell subsets (TM and EM) are preferentially targeted for infection by C-HIV Envs in vitro, and that tropism remained consistent during progression from acute to chronic disease.

## Results

### Establishment of a longitudinal bank of C-HIV Envs

To understand how virus tropism for different memory CD4^+^ T cell subsets changes during a C-HIV infection, we obtained longitudinal *env* clones (Additional file 1: Table S1) from five C-HIV-positive individuals enrolled in the CAPRISA 002 Acute Infection Study [41]. Samples were obtained at less than 2 months (referred to as T0), 1 year (T1) and 3-years post-infection (T3). The estimated duration of infection, CD4 T cell count and plasma viral load for each time point sampled are shown in Table 1. The median age of participants at enrolment was 28 years (range: 24–37), and the median estimated duration of infection was 39 days (range: 14–55 days) post-infection. CD4 T cell counts were reduced at T3 compared to T0 for all five participants (reduced by 165 to 423 cells/µl). Plasma viral load had reduced in three participants (CAP177, CAP255 and CAP257; decreasing by: 0.01 to 1.23 log_10_ copies per ml) and increased in two (CAP88 and CAP228; increased by: 0.59 to 0.69 log_10_ copies/ml) by T3 compared to T0.

**Table 1:** Clinical characteristics of subjects enrolled in the CAPRISA acute infection cohort

Participant ID	Age	Acute (T0)			1 year (T1)			3 years (T3)			Δ pVL	Δ CD4 count
		pVL	CD4 count	Time (days)	pVL	CD4 count	Time (days)	pVL	CD4 count	Time (years)		
CAP88	24	4.46	963	37	5.00	397	400	5.15	627	3	0.69	− 336
CAP177	37	5.55	435	14	4.25	381	351	4.68	270	3	− 0.87	− 165
CAP228	37	3.57	867	46	3.18	642	360	4.16	481	3	0.59	− 386
CAP255	27	5.29	772	55	4.26	397	357	4.06	349	3	− 1.23	− 423
CAP257	28	4.75	689	39	5.25	291	369	4.74	274	3	− 0.01	− 415

Plasma viral load (pVL) is shown in log_10_ copies/ml, CD4 count is represented as cells/μl, and time represents the time since estimated primary infection. Δ represents the change in pVL or CD4 count between acute and 3-years post-infection (3-years post-infection minus acute)

### Env cloning and genotypic characterisation

**Fig. 1:**
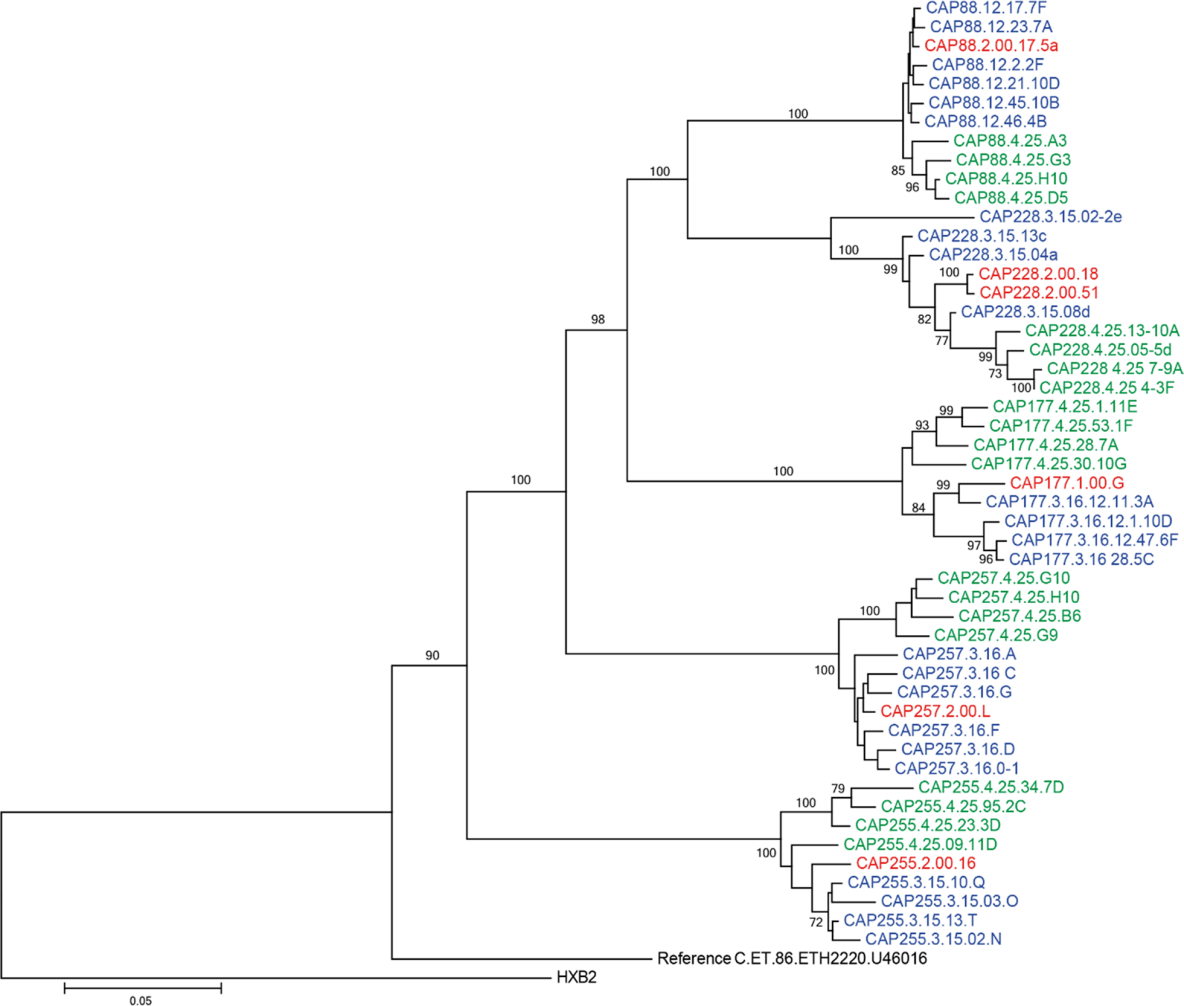
Phylogenetic analysis of the intra- and inter-patient relationship between clinical C-HIV Envs. The phylogenetic tree was generated with 52 *env* nucleotide sequences of the *Acc65I*–*BamHI* region (corresponding to amino acid positions 6348–8478 of HXB2). This tree confirms that there was no inter-subject mixing of *env* sequences and that all sequences were subtype C as they clustered with an unrelated C-HIV isolate (C.ET.86.ETH2220.U46016) instead of the out-group sequence HXB2. Red font labels indicate sequences isolated at enrolment (acute infection), blue labels indicate sequences isolated at 1-year post-infection, green labels indicate sequences isolated at 3-years post-infection and black labels indicate control sequences. The scale represents number of nucleotide substitutions per site and numbers on branches indicate bootstrap values

A total of 50 C-HIV *envs* from five participants were cloned into the pSVIII-Env expression vector using the primers indicated in Additional file 1: Table S2; 1–2 clones at T0, 4–6 clones at T1 and four clones at T3 for each participant (Table 2). Sequence and phylogenetic analysis of C-HIV *envs (Acc65I*–*BamHI* region corresponding to amino acid positions 6348–8478 of HXB2) showed no inter-participant mixing of sequences (Fig. 1). Furthermore, all generated C-HIV sequences were more closely related to a reference C-HIV isolate (C.ET.86.ETH2220.U46016) than HXB2 (subtype B HIV-1), confirming these sequences were C-HIV. We also observed compartmentalisation between T1 *envs* and T3 *envs* in all participants, suggesting viral evolution in e*nv* had occurred between the two-time points (Fig. 1).

**Table 2:** Characterisation of C-HIV Env phenotypes

Patient ID	Acute (T0)	1 year (T1)	3 years (T3)	Summary of phenotypes
CAP88	R5 (n = 1)	R5 (n = 3) R5/X4 (n = 3)	R5 (n = 4)	R5/X4
CAP177	R5 (n = 1)	R5 (n = 3)	R5 (n = 4)	R5
CAP228	R5 (n = 2)	R5 (n = 4)	R5 (n = 3)	R5
CAP255	R5 (n = 1)	R5 (n = 3)	R5 (n = 4)	R5
CAP257	R5 (n = 1)	R5 (n = 5)	R5 (n = 3)	R5

R5: CCR5-using, X4: CXCR4-using

**Fig. 2:**
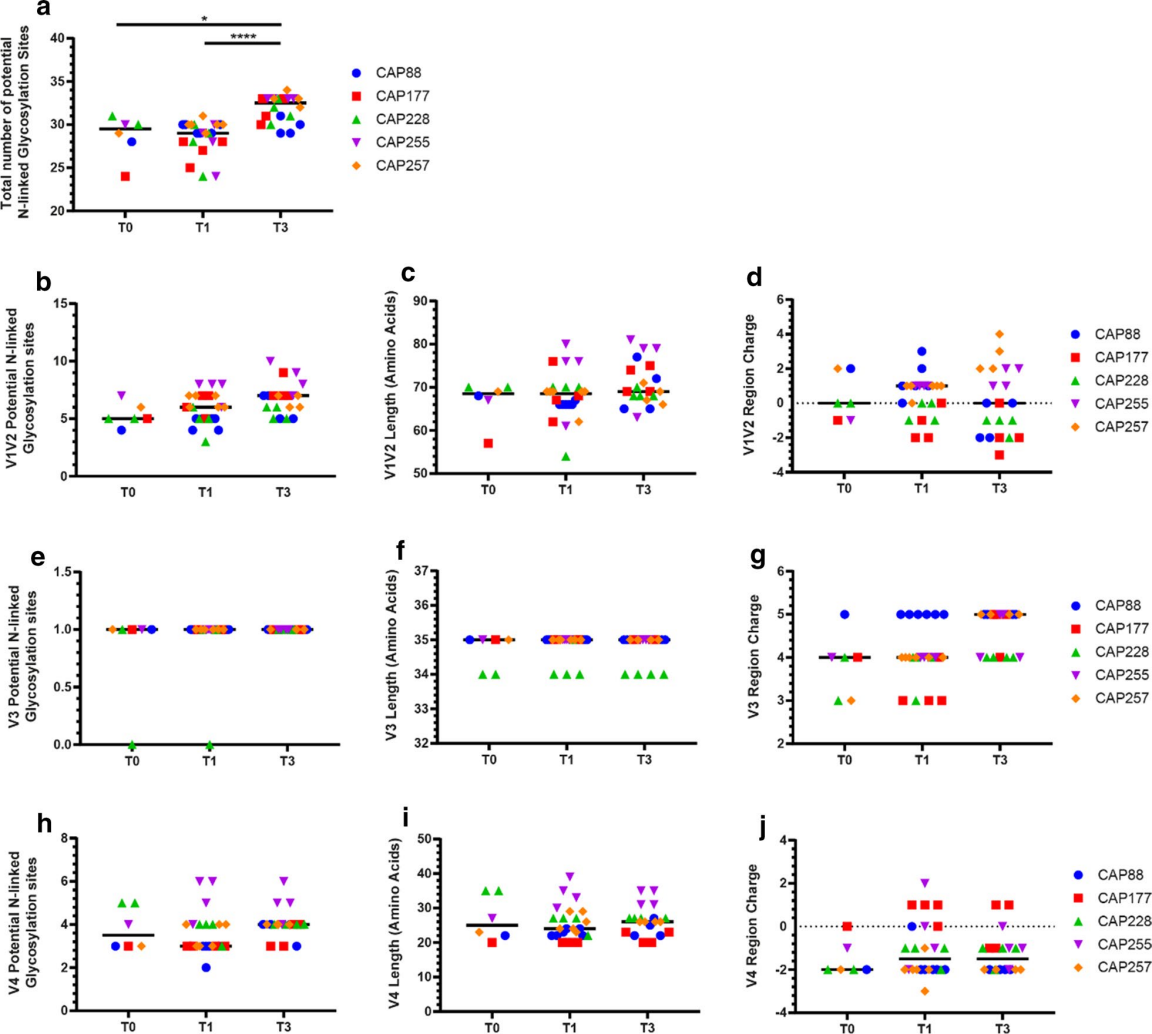
Genotypic characteristics of longitudinal C-HIV Envs during natural infection. **a** The number of potential N-linked glycosylation sites (PNLGS) within the *Acc65I*–*BamHI* fragment of Env stratified by time point (T0, T1, T3) and Env donor indicated as follows; CAP88 (blue circles), CAP177 (red squares), CAP228 (green triangles), CAP255 (purple inverted triangles) and CAP257 (orange diamonds). The **b** PNLGs, **c** length in amino acids and **d** net charge of the V1V2 region within Envs. **e** PNLGs, **f** length (amino acids) and **g** net charge within the V3 region of C-HIV Envs. **h** PNLGS, **i** length (amino acids) and **j** net charge within the V4 region of C-HIV Envs. Black lines represent the median within each time point. For net charge graphs (**d**, **g**, and **j**), the dotted line represents a net charge of 0. Comparisons were made using a Kruskal–Wallis test with Dunn’s post hoc test for multiple comparisons, *p < 0.05, ****p < *0.0001*

Next, we explored genotypic changes in *envs* over the first 3 years of untreated infection. We analysed Env amino acid length, charge and potential N-linked glycosylation sites (PNLGS) within the variable loops of Env (V1/V2, V3 and V4). We found that total Env PNLGS had increased at T3 (median: 32.5, range: 29–34) compared to T1 (median: 29, range: 24–31) and T0 (median: 29.5, range: 24–31) (Fig. 2a), suggesting that sites of glycosylation increased over time, consistent with previous subtype B and C studies [15, 42, 43]. We found no change in V1/V2 median PNLGs, length and charge at T3 compared to T1 and T0 (Fig. 2b–d), although the mean showed a trend for increased V1/V2 length at T3 compared to T0 and T1 (70.8 vs 66.8 and 68.1 respectively). No differences were observed in V3 PNLGs, length or charge (Fig. 2e–g). Coreceptor usage was predicted using two prediction algorithms, Phenoseq and CoReceptor USage prediction for HIV-1 (CRUSH) [44–46]. These algorithms were preferred to geno2pheno due to their improved sensitivity for C-HIV sequences [44–46]. Coreceptor prediction showed all C-HIV *envs* were predicted to be CCR5-using (Additional file 1: Table S3). Furthermore, sequence analysis of V3 regions within each participant revealed limited alterations in amino acid sequence over 3 years (Additional file 1: Figure S1), consistent with previous studies showing limited V3 alterations in C-HIV Envs [47, 48]. We found no change in V4 PNLGs, length or charge (Fig. 2h–j). Overall, our genotypic characteristic analysis of clinical C-HIV *envs* demonstrated few alterations within the variable regions over time. However, we observed an increase in total Env PNLGs over time, which may affect virus tropism for different memory CD4^+^ T cell subsets. Furthermore, our data shows that not one single variable region showed significant increases in PNLGs, suggesting multiple regions within Env were likely contributing to the increase in total PNLGs.

### Functional characterisation and coreceptor usage of C-HIV Envs

To test C-HIV Env functionality and coreceptor usage, we pseudotyped luciferase reporter viruses with 50 C-HIV Envs [49–53]. Pseudoviruses were used in single-round entry assays using NP2 cells stably expressing CD4 and either CCR5 or CXCR4 [54]. We found 45 pseudoviruses generated high titres to permit functional phenotype assessment, while five pseudoviruses demonstrated infectivity too low to appropriately phenotype and were excluded from the study (Additional file 1: Table S3). Functional pseudoviruses were assessed for coreceptor usage by converting the luciferase signal (RLU, relative luciferase units) into an arbitrary score for entry via each coreceptor (‘strong’; +++, ‘medium’; ++ or ‘weak’; +). We found all functional pseudoviruses mediated entry into NP2/CD4/CCR5 cells (Table 2, Additional file 1: Table S3). Additionally, three viruses utilised CXCR4 for entry into NP2/CD4/CXCR4 cells (CAP88.12.17.7F, CAP88.12.23.7A, and CAP88.12.46.4B); however, infectivity via CXCR4 was weak (Table 2, Additional file 1: Table S3). Interestingly, an inspection of the V3 loop amino acid sequences of these three Envs demonstrated no alterations compared to the T0 Env, which remained exclusively CCR5-using (Additional file 1: Figure S1a).

To confirm CXCR4 usage by CAP88.12.17.7F, CAP88.12.23.7A, and CAP88.12.46.4B, we conducted viral inhibition assays using the CXCR4 antagonist AMD3100. Our results showed that 5 µM AMD3100 strongly inhibited entry of these viruses compared to infection levels in the absence of drug (Additional file 1: Figure S2). In contrast, infection by CXCR4-using HXB2 and dual-tropic 89.6 was completely inhibited (Additional file 1: Figure S2). This data suggests that these viruses can mediate infection through CXCR4 despite demonstrating no alterations in V3 sequence (Additional file 1: Figure S1).

### Tropism for memory CD4^+^ T cell subsets

To determine memory CD4^+^ T cell subset tropism of C-HIV Envs and whether tropism changes over the first 3 years of infection, we used an in vitro multi-colour flow cytometry assay [32, 38, 39]. Env-pseudotyped GFP-expressing reporter viruses were generated with an NL4.3 backbone [55] and titrated on TZMbl cells. We found 78% (39/50) of Envs produced high titre GFP-pseudoviruses to enable assessment of CD4^+^ T cell infection. These pseudotyped reporter viruses were used to infect CD4^+^ T cells isolated from HIV-seronegative donors. Infection of CD4^+^ T cell subsets was investigated in two ways; as a percentage of total infected CD4^+^ T cells to understand the contribution of each subset to the pool of infected CD4^+^ T cells (Additional file 1: Figure S3), and as a percentage of total cells infected within the respective subset to understand infectivity of each CD4^+^ T cell subset (Additional file 1: Figure S4).

**Fig. 3:**
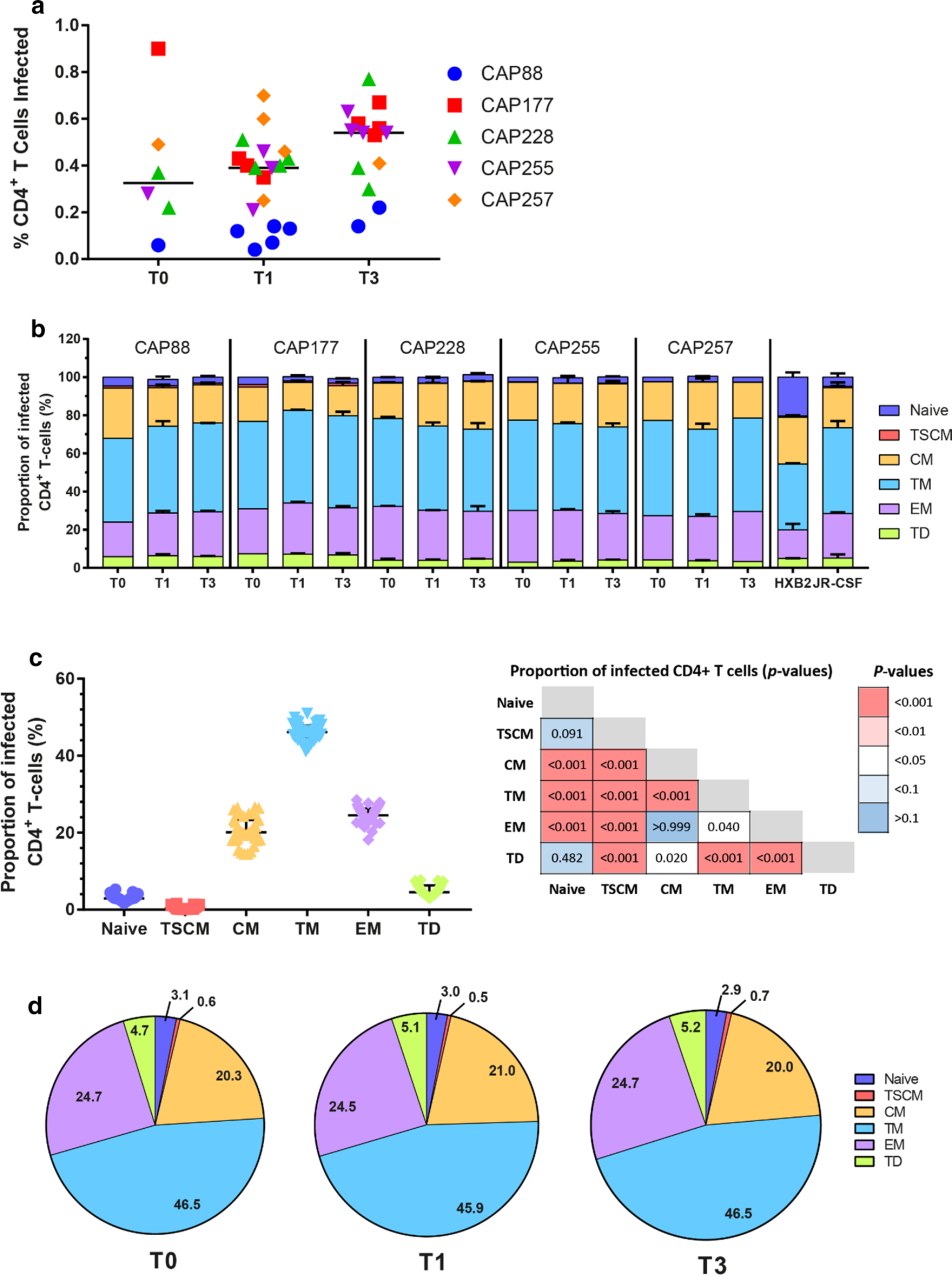
Transitional memory and effector memory cells were most frequently infected by C-HIV Envs. **a** Each data point represents the percentage of infected CD4^+^ T cells with one pseudovirus (averaged from four independent seronegative blood bank donors). The Env donor is indicated as follows; CAP88 (blue circles), CAP177 (red squares), CAP228 (green triangles), CAP255 (purple inverted triangles) and CAP257 (orange diamonds). Black lines represents the median of all pseudoviruses within each time point. Comparisons were made using a Kruskal–Wallis test with Dunn’s post hoc test for multiple comparisons. **b** Stacked bar graphs represent the contribution of each T cell subset to the pool of infected CD4^+^ T cells. Values represent the median percentage of infected CD4^+^ T-cells (averaged across four HIV-seronegative PBMC donors) that belong to the indicated subset [naïve; dark blue, T stem cell memory (TSCM); red, central memory (CM); yellow, transitional memory (TM); light blue, effector memory (EM); purple and terminally differentiated (TD); green], and are stratified by participant and time point. Error bars represent the interquartile range. **c** Dot plot representing the proportion of each T cell subset contributing to the total pool of infected cells for all Env-pseudoviruses. Each point represents a single virus averaged across four seronegative donors, lines represent median and error bars represent interquartile range. Comparisons were made using a Kruskal–Wallis test with Dunn’s post hoc test for multiple comparisons and are shown in the table. **d** Pie charts represent the proportion each CD4^+^ T cell subset contributes to the total pool of infected CD4^+^ T cells for each time point. Each pie slice represents the average of all viruses from T0, T1 and T3 respectively for the indicated T cell subset (naïve; dark blue, TSCM; red, CM; yellow, TM; light blue, EM; purple and TD; green)

We first analysed the percentage of total CD4^+^ T cells productively infected (CD3+GFP+) across all time points and participants and observed no differences in infection efficiency between time points (Fig. 3a).

Next, we assessed the contribution of each T cell subset to the total pool of productively infected cells. We used two control viruses, JR-CSF (CCR5-using) and HXB2 (CXCR4-using) to confirm that differential coreceptor usage leads to differences in memory CD4^+^ T cell tropism (Fig. 3b). Our results demonstrated that HXB2 infected a higher proportion of naïve cells (20% of total cells infected) than JR-CSF (4% of total cells infected), which is consistent with our previous studies [32, 38]. Furthermore, HXB2 showed a reduced proportion of infected TM and EM cells (34.6% and 15% respectively) compared to JR-CSF (45.1% and 23.3% respectively).

We next assessed whether Envs from untreated C-HIV infection demonstrate altered cellular tropism for different memory CD4^+^ T cell subsets over time. First, we found that TM, EM and CM cells constituted the majority of infected CD4^+^ T cells (medians: 46.1%, 24.5% and 20.1% of total infected cells respectively; Fig. 3c). In contrast, naive, TSCM and TD cells contributed low proportions (3%, 0.6% and 5%) of total infected cells. When we stratified the proportion of each subset infected by participant and time of Env sampling, we found no substantial variation in cellular tropism for memory CD4^+^ T cell subsets across T0, T1 or T3 for any participant (Fig. 3b and Additional file 1: Figure S5). We did not perform statistical analyses on each participant data set between time points due to limited virus numbers (n = 1–5 Envs per time point). When we compared CD4 T cell subsets infected across the different participants, we found that Env-pseudoviruses containing CAP88 or CAP177 Envs showed a more robust infection of TSCM cells (range: 0.72–1.56% of total infected cells; Additional file 1: Figure S5b) compared to CAP228, CAP255 or CAP257 Envs (range 0.13–0.33% of total infected cells). In addition, we found no difference in subset tropism between CAP88 T1 Envs that infected NP2/CD4/CXCR4 cells (R5/X4 using n = 3) and those that did not (R5 using n = 3), suggesting that the level of CXCR4 usage by these viruses was too low to detect a difference in infection of CD4^+^ T cell subsets. When we combined the data from each participant, we observed no significant alterations in T cell tropism over the three-time points (Fig. 3d).

**Fig. 4:**
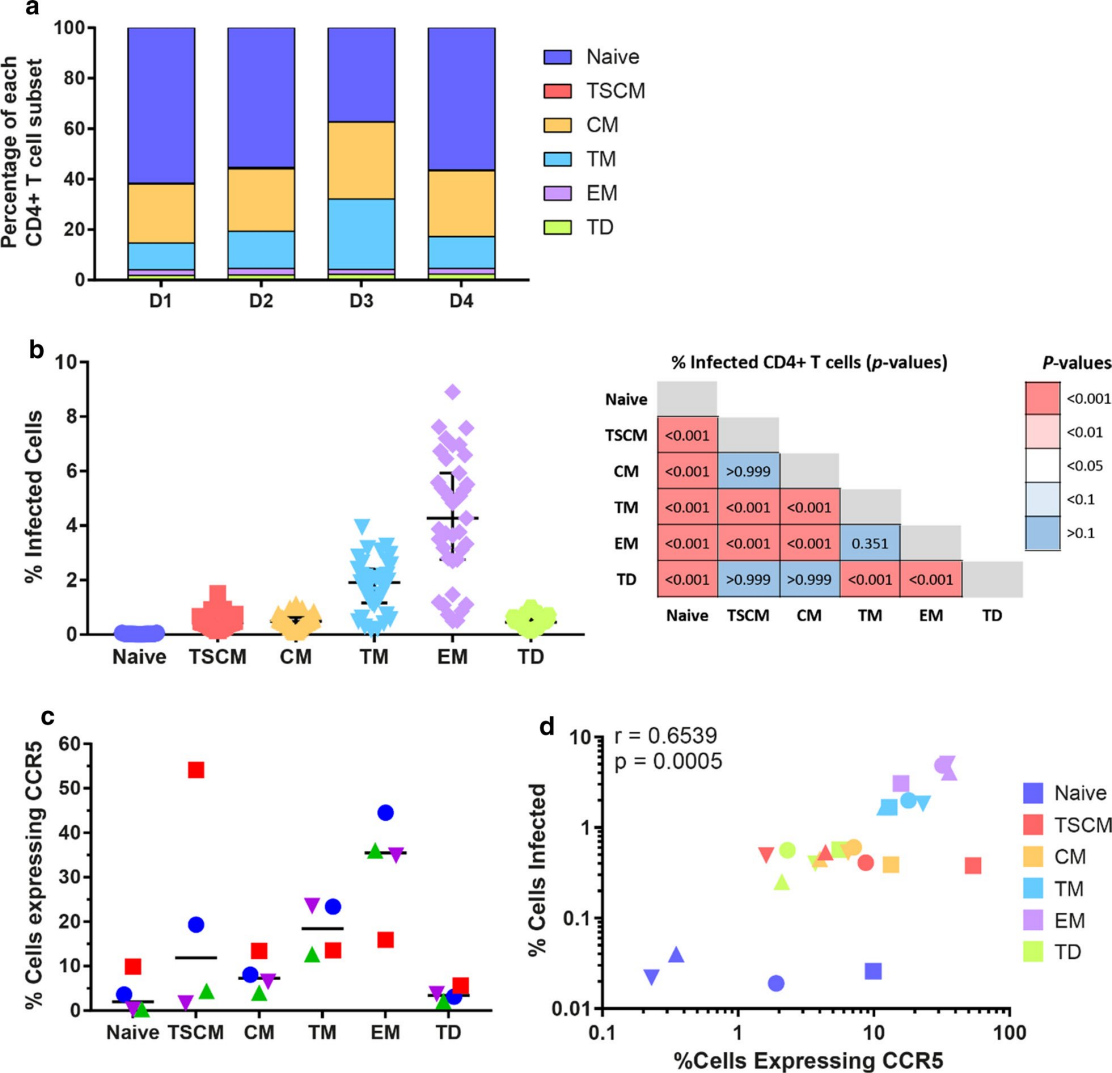
Effector memory cells were preferentially infected and expressed more CCR5 compared to other subsets. **a** Stacked bar graphs represent the contribution of each CD4^+^ T cell subset to the total CD4^+^ T cell pool before infection, stratified by HIV-seronegative donor. Subsets are denoted as follows: naïve; dark blue, T stem cell memory (TSCM); red, central memory (CM); yellow, transitional memory (TM); light blue, effector memory (EM); purple and terminally differentiated (TD); green. **b** The percentage of infected cells per CD4^+^ T cell subset 72 h after inoculation with GFP reporter viruses. Each data point represents one virus, where the percentage of cells expressing GFP is averaged across four HIV-seronegative donors. Black lines represent the median of each virus, while the error bars represent interquartile range. Comparisons were made using a Kruskal–Wallis test with Dunn’s post hoc test for multiple comparisons and are shown in the table. **c** CCR5 expression on the indicated T cell subsets after CD4 T cell isolation and before infection. Each data point represents percentage of cells expressing CCR5 from a single HIV-seronegative donor with black lines representing median. **d** Spearman correlation between percentage of each T cell subset expressing CCR5 before infection and the percentage of cells infected within each T cell subset. Each data point represents a single donor, with the percentage of cells infected averaged from n = 39 pseudovirus infections. Circles, squares, triangles and upside-down triangles represent donors 1, 2, 3 and 4 respectively, while symbol colour represents T cell subset (naïve; dark blue, TSCM; red, CM; yellow, TM; light blue, EM; purple and TD; green)

Although TM and EM subsets made up the majority of infected cells, they only contributed a minor fraction of the total CD4^+^ T cell pool in uninfected cells (TM range: 10.7–27.8% and EM range: 2–2.6%, Fig. 4a). High levels of infection but low proportions of these subsets in the total T cell pool suggests that they were more susceptible to infection compared to other subsets. Therefore, we chose to determine the infectivity of each CD4^+^ T cell subset by analysing the percentage of cells infected within each subset. For this analysis, we first identified each CD4^+^ T cell subset based on surface marker expression before assessing GFP positivity (Additional file 1: Figure S4). This analysis demonstrated preferential infection of EM cells (median: 5.4% EM cells infected, range: 2.7–8.9%) and TM cells (median: 2.2% TM cells infected, range: 1.1–3.9%), compared to all other subsets (Fig. 4b). Consistent with the increased proportion of EM infection, coreceptor expression analysis on each T cell subset prior to infection revealed that more EM cells expressed CCR5 (35.5%) compared to other subsets (Fig. 4c). Additionally, more TM cells expressed CCR5 (18.5%) compared to naive, CM and TD cells (Fig. 4c). We observed a similar trend in the intensity of CCR5 expression across the different subsets (Additional file 1: Figure S6). Moreover, we observed a highly significant association between the expression of CCR5 prior to infection in a given subset and the percentage of cells infected (*p *= 0.0005, Fig. 4d). Overall, our data suggests that preferential infection of memory CD4^+^ T cell subsets by CCR5-using viruses is associated with the relative expression of CCR5 on each subset.

## Discussion

HIV-1 infects a broad range of CD4^+^ T cell subsets with different phenotypic properties, including coreceptor expression [32, 33] and cellular activation status [30]. Given that C-HIV is the most prevalent subtype of HIV-1 globally, we sought to determine the cellular tropism of C-HIV Envs sampled from the first 3 years of untreated infection for different memory CD4^+^ T cell subsets. Despite considerable evolution of *envs* within each participant, including the addition of PNLGS, all C-HIV Envs remained CCR5-using with three using CXCR4 weakly in NP2/CD4/CXCR4 cells. We found that TM and EM cells were more frequently infected than other subsets in vitro, which was associated with higher relative expression of CCR5 on these subsets. Our data showed no significant change in CD4^+^ T cell subset tropism in clones sampled from the first 3 years of untreated C-HIV infection. Given that we sampled longitudinally Envs from untreated people living with C-HIV, our findings likely recapitulate viral tropism properties of viruses found within individuals during the progression from acute to chronic HIV-1 infection.

When we analysed the percentage of infected cells per subset, we found EM cells were preferentially infected compared to other subsets. This preferential infection of EM cells is likely mediated through the higher expression of CCR5 on these cells [32, 33, 56]. However, while our data suggests that infection of different memory CD4^+^ T cell subsets is reliant on the level of CCR5 expression, we cannot rule out other factors known to contribute to viral tropism including enhanced CD4 usage or fusogenicity [57]. Our findings are consistent with those from Parrish et al., which found viruses pseudotyped with Envs from C-HIV transmitted/founder viruses predominantly infected EM cells (~ 70% of total infected CD4^+^ T cells) in vitro [18]. The lower proportion of infected EM cells in our study (~ 25% of total infected CD4^+^ T cells) can be ascribed to by (i) the separation of TM and EM cells using CD27 in our study and (ii) the stimulation of CD4^+^ T cells prior to infection with anti-CD3 and anti-CD28 in the Parrish et al. study, which is likely to create a more differentiated memory CD4^+^ T cell population with more cells expressing CCR5 [40, 58].

Acute HIV-1 infection results in immune activation [59] that may drive the formation of a more differentiated CD4^+^ T cell pool and thus more cells expressing CCR5 [60, 61]. Moreover, a study by Douek et al. found that HIV-1-specific CD4^+^ T cells contained more HIV-1 viral DNA per million cells than other memory CD4^+^ T cells during acute infection [62]. Therefore, HIV-1-driven immune activation and CD4^+^ T cell differentiation could drive HIV-1 dissemination through providing more susceptible CD4^+^ T cell targets.

Previous studies have demonstrated that TSCM and CM subsets are highly stable reservoirs during HIV-1 infection due to their capacity for self-renewal through cellular proliferation [34–36]. As such, we were interested in the ability of C-HIV Env pseudoviruses to mediate infection in these cell types and whether infectivity changed over disease progression. Our data shows that CM cells were readily infected by C-HIV Envs sampled from acute to chronic disease stages, making up ~ 20% of productively infected CD4^+^ T cells. Our findings suggest that viral reservoirs of latently infected cells could be established early through the direct infection of CM cells. Given that CM cells represent 23–30% of the total CD4^+^ T cell pool, this represents a potentially significant source for the establishment of the viral reservoir. Consistent with this, a study of individuals infected with subtype AE found that CM cells were infected at similar frequencies to TM and EM subsets during acute infection [63].

Contrary to our hypothesis, we did not see an increase in CM infection by viruses at T3 compared to earlier time points, suggesting C-HIV Envs did not improve CCR5 utilisation during progression from acute to chronic disease in these participants. Additionally, we found TSCM cells made up less than one per cent of productively infected cells. The low level of infection in TSCM cells is likely a result of their low proportion in the blood and a low surface expression of CCR5 [32, 33]. Overall, our data shows that while C-HIV Envs demonstrated consistent low levels of TSCM and CM infection, the ability (particularly by T0 Envs) to infect these subsets in vitro suggests a mechanism by which long-lived genetically stable reservoirs may be established.

Glycan sites on the surface of Env play an important role in evasion of neutralising antibody responses throughout HIV-1 infection [64, 65]. The addition or removal of glycan sites within Env can influence the infectivity and sensitivity of viruses to neutralising antibodies [64–67]. Indeed, we observed an increase in N-linked glycosylation sites within Envs over time, suggesting subtle structural changes occurred in Envs over the course of infection. Furthermore, CAP177, CAP228, CAP255 and CAP257 developed neutralising antibody responses with varying levels of breadth within 3 years of infection [68]. Interestingly, we found these participants showed increases in Env PNLGs from T1 to T3, suggesting that increased PNLGs may be driven by viral escape from neutralising antibody responses. In contrast, CAP88 showed no change in PNLGs over time and did not develop a broadly neutralising antibody response. Structural alterations in Env in response to neutralising antibody responses have been well documented for most participants in this study [69–72]. For instance, CAP257 developed multiple broadly neutralising antibody responses over the first 3 years of infection to the V2 region and CD4 binding site of Env, with subsequent mutations in the viral protein driving escape from these responses [71]. Furthermore, CAP177 developed neutralising antibodies targeting the V3C3 region within the first 6 months of infection, with viral escape leading to increased V1 loop length and glycan rearrangement from position N334 to N332 [69, 72]. This N332 glycan subsequently reverted to an N334 glycan by 2 years post-infection to escape an N332-targeting neutralising antibody response [69, 72]. Evidently, these structural alterations that occurred in Envs as a counter measure to neutralising antibody responses, were insufficient to impact CD4^+^ T cell subset tropism.

In our study we acknowledge that our CD4^+^ T cell subset tropism assay only detects productive infection through GFP expression. Not only do we not detect latent infection of each subset, but there may be barriers between viral entry and GFP expression that could block reporter expression and lead to an underestimation of the level of productive infection in each subset. In addition, more differentiated memory cells demonstrate a progressive loss in DNA methylation [73], and more acetylated H3/H4 histones compared to less differentiated memory cells [74]. Therefore, we cannot rule out that host epigenetics may contribute to a reduction in detection of productive infection in less differentiated CD4^+^ T cell subsets in our assay. However, Tabler et al. showed similar ratios of productive infection to viral entry across naïve, CM and EM cells using a CCR5-using reporter virus [33]. Latent infection in this model could be assessed through assaying total HIV-1 DNA per million cells or through using a dual-fluorescence reporter virus construct that allows quantification of latent and productive infection using two different fluorescent reporter genes [4, 75]. Moreover, the total number of virions that fuse with cells and do not establish a productive or latent infection (referred to as an abortive infection) could be assessed through using a Vpr-Blam and GFP expressing reporter virus as described by Tabler et al. [33]. It is also possible that our sampling of the first 3 years of infection was insufficient to observe alterations within Envs that significantly change cellular tropism for different CD4^+^ T cell subsets. Well characterised alternative Env phenotypes such as coreceptor switching to CXCR4 usage [21–23] or the development of macrophage-tropic Envs [53, 76–78] occur during late stages of disease when memory CD4^+^ T cells are considerably depleted (CD4^+^ T cell counts < 200 cells/μl). Therefore, future studies assessing changes in Env phenotypic properties may benefit from sampling over more than 3 years.

## Conclusions

In summary, our data demonstrates that despite viral evolution over the first 3 years of C-HIV infection, CD4^+^ T cell tropism of C-HIV Envs was not significantly altered in vitro. Instead, we found C-HIV Envs preferentially infected more differentiated cell types, particularly EM cells. This preferential infection was likely mediated through a higher proportion of EM cells expressing CCR5 compared to less differentiated subsets. Furthermore, we found that while preference for infection of cells implicated as long-lived HIV-1 reservoirs (TSCM and CM cells) was low, C-HIV Envs isolated from acute to chronic disease progression could mediate infection in these subsets in vitro. Our data provide novel insights into the cellular tropism of longitudinal C-HIV Envs and the establishment of viral reservoirs from early to chronic stages of infection.

## Methods

### Ethics statement

Five participants were selected from the Centre for the AIDS Programme of Research in South Africa (CAPRISA) 002 acute infection cohort [41], which commenced in 2004 in Durban and Vulindlela, South Africa. All study participants provided written informed consent prior to study enrolment. The study protocol was approved by the Ethics Committee of the Nelson R. Mandela School of Medicine (FWA #00000678, primary) as well as through local ethics committees at the University of the Witwatersrand and the University of Cape Town, and was conducted according to the Declaration of Helsinki and ICH/GCP guidelines.

### Participants

Enrolment of study participants has been previously described [41]. Diagnosis of infection, as well as the collection of clinical characteristic data including CD4 count, plasma viral load, and the estimated time of infection, have been described previously [68, 79].

### Cell lines

293T cells, TZMbl cells [80], NP2/CD4/CXCR4 and NP2/CD4/CCR5 cells [54] were maintained in Dulbecco’s Modified Eagle Medium (DMEM) supplemented with 10% (vol/vol) fetal calf serum (FCS), 100 μg/ml of penicillin and streptomycin and 100 μg/ml l-glutamine. CD4 selection in NP2 cells was maintained with 500 μg/ml of G418 and chemokine receptor expression (CXCR4 or CCR5) was maintained with 1 μg/ml of puromycin. PBMCs and CD4^+^ T cells were maintained in RPMI 1640 supplemented with 10% (vol/vol) FCS, 100 μg/ml of penicillin and streptomycin and 100 μg/ml l-glutamine.

### Env amplification and cloning

C-HIV *envs* sequences were amplified from plasma samples using single genome amplification (Additional file 1: Table S1) and cloned into the pcDNA3.1 expression vector [69–71, 79]. For efficient Env pseudotyping onto HIV-1 particles, we used a PCR reaction to generate *Acc65*I–*BamH*I flanked *env* fragments (corresponding to amino acid positions 6348–8478 of HXB2) using Platinum Taq HiFi (Invitrogen, USA) and the primers described in Additional file 1: Table S2. PCR products were cloned into the pSVIII-Env expression plasmid using *Acc65*I and *BamH*I restriction digest sites as described previously [22, 50, 53, 78, 81–85]. *Envs* (n = 15) that were unable to be effectively cloned were subsequently synthesised by Genescript (Piscataway, New Jersey, USA). All *envs* were sequenced using Sanger sequencing to confirm fidelity. *Envs* shown to be functional and able to support HIV-1 entry into NP2/CD4/CCR5 or NP2/CD4/CXCR4 cells when pseudotyped onto luciferase reporter viruses were included in the study [22, 50, 53, 78, 82–85]. Functional *env* clone accession numbers are listed in Additional file 1: Table S1.

### Env sequencing and phylogenetic analysis

*Env* sequences were trimmed to the *Acc65I* to *BamHI* region (positions 6348–8478 relative to HXB2) and aligned using ClustalW in CLC Main Workbench 8.0. (Qiagen, Hilden, Germany). A maximum-likelihood tree was generated using MEGA7 [86]. Evolutionary history was inferred using the Maximum-likelihood method based on the General Time Reversible model in addition to a discrete gamma distribution to model evolutionary rate [87], and 100 bootstrap replicates were generated for variance estimation. This model was selected as the most appropriate for our dataset by the Los Alamos National Laboratory FindModel tool (https://www.hiv.lanl.gov/content/sequence/findmodel/findmodel.html). The number of potential N-linked glycosylation sites was determined using N-glycosite (http://www.hiv.lanl.gov/content/sequence/GLYCOSITE/glycosite.html/). Variable region length and charge were calculated using Microsoft Office Excel 2016. Coreceptor usage of each C-HIV *env* was predicted using two coreceptor prediction programs, Phenoseq (http://tools.burnet.edu.au/phenoseq/) [44, 45], and CRUSH (http://ares.tamu.edu/CRUSH/) [46], both of which use the V3 loop as input data.

### Production and titration of Env-pseudotyped reporter viruses

Env-pseudotyped luciferase reporter viruses were produced by transfection of 293T cells with plasmids pCMVΔP1ΔenvpA, pHIV-1Luc, and pSVIII-Env using Lipofectamine 2000 (Invitrogen, USA) at a ratio of 1:3:1, as described previously [22, 50, 53, 78, 82–85]. Supernatants were harvested 48 h later, filtered through 0.45 μM-pore size filters, and stored at − 80 °C. Virus stocks were quantified based on corrected luciferase units in NP2/CD4/CCR5 and NP2/CD4/CXCR4 cells, as described previously [22, 39, 50, 84, 85, 88, 89]. Env-pseudotyped GFP reporter viruses were produced by transfecting 293T cells with pNL4‐3Env‐GFP and pSVIII-Env plasmids using Lipofectamine 2000 (Invitrogen, USA) at a ratio of 4:1, as previously described [32, 38, 39]. Supernatants were harvested 48 h later and filtered through 0.45 μm filters. Viruses were concentrated by Lenti-X concentrator (Clontech, California, USA) using the manufacturer’s protocol, and stored at − 80 °C. The 50% tissue culture infectious doses (TCID50) of Env-pseudotyped GFP reporter virus stocks was determined by titration in TZMbl cells, as previously described [32, 38, 39].

### HIV-1 Env Co-receptor usage

The ability of Env-pseudotyped luciferase reporter viruses to use CCR5 and/or CXCR4 was determined by single-round entry assays using NP2/CD4/CCR5 or NP2/CD4/CXCR4 cells, which stably express CD4 together with CCR5 or CXCR4, as described previously [50]. Briefly, 10,000 cells were inoculated with fivefold serial dilutions of virus for 16 h at 37 °C. Cell media was replaced on cells and incubated for a further 48 h at 37 °C. HIV-1 entry was measured by quantification of luciferase activity (RLU) in cell lysates (Promega, California, USA), according to the manufacturers’ protocol. Luminescence was measured using a CLARIOStar microplate reader (BMG Labtech, North Carolina, USA). Background luminescence as determined by the average luciferase activity of uninfected cells was subtracted from luminescence values for each sample.

### HIV-1 inhibition assays

HIV-1 inhibition assays in NP2/CD4/CXCR4 cells were performed, as previously described [51, 84, 85]. Briefly, 10,000 cells were seeded into flat-bottom 96-well plates. The CXCR4 antagonist AMD3100 was resuspended in dimethyl sulfoxide (DMSO). Prior to infection, cells were incubated with 5 μM of AMD3100 for 30 min at 37 °C. Untreated controls were maintained in 0.1% (vol/vol) DMSO. Cells were inoculated with approximately 100,000 luciferase units of Env-pseudotyped luciferase reporter viruses (as determined by titration in NP2/CD4/CXCR4 cells) and incubated at 37 °C for 72 h. The level of HIV-1 entry was measured by luciferase activity in cell lysates as described above. Background luminescence was quantified in uninfected cells and subtracted from all samples. The amount of luciferase activity in cells treated with an inhibitor was expressed as a percentage of that in untreated cells [51, 84, 85].

### Quantifying HIV-1 infection of CD4^+^ T cells subsets

To determine CD4^+^ T cell subset tropism, we utilised an in vitro multi-parameter flow cytometry-based infection assay, described previously [32, 38, 39]. Briefly, peripheral blood CD4^+^ T-cells were isolated from four HIV-seronegative donors using a CD4 T cell isolation kit (Miltineyi Biotech, Bergisch Gladbach, Germany), with an average purity of 92%. Each donor was treated as an independent infection and 1.5 million CD4^+^ T cells were spinoculated (1200 g for 2 h) with 3000 TCID_50_ single-round Env-pseudotyped GFP reporter viruses in 96-well V-bottom plates. GFP reporter viruses pseudotyped with subtype B Envs JR-CSF (CCR5) and HXB2 (CXCR4) were used as controls [32, 38, 39]. Cells were transferred to 48 well plates and incubated for 3 days at 37 °C prior to staining with the following fluorochrome labelled antibody panel: CD4 FITC (Clone: RPA-T4, BD Biosciences #555346), CD122 PerCP-eF710 (Clone: TU27, eBioscience # 46-1228-42) or CD122 PerCP-eF710 (Clone: 46-1229, eBioscience #46-1229, discontinued), CCR7 AF647 (Clone: 3D12, BD Biosciences # 557734), CD3 APC-Cy7 (Clone: SK7, BD Biosciences #341090), CD45RO eF450 (Clone: UCHL1, eBioscience #48-0457), Fixable viability dye eF506 (eBioscience #65-0866), CD95 PE-CF594 (Clone: DX2, BD Biosciences #562395), CD27 PE-Cy7 (Clone: M-T271, BD Biosciences # 560609), CCR5 PE (Clone: 2D7, BD Biosciences #555993), and CXCR4 PE-Cy5 (Clone: 555975, BD Biosciences #555975). Cells were fixed for 1 h in paraformaldehyde (4% wt/vol), then washed and resuspended in filtered FACS buffer (filtered PBS with 2% FCS).

We collected 700,000–1,000,000 events per donor using a BD LSR Fortessa flow cytometer (BD Biosciences, California, USA) and analysed with FlowJo version 10 software (Treestar, Oregon, USA). OneComp ebeads (eBiosciences #01-1111) were used with flow cytometry antibodies as compensation controls. The percentage of HIV-1 infected cells in each CD4^+^ T cell subset was determined by excluding doublets and dead cells, gating on CD3+GFP+ expressing cells and then phenotyping subsets as follows; naïve (CD45RO−CCR7+), TSCM (CD45RO−CCR7+CD95+CD122+), TD (CD45RO−CCR7−), CM (CD45RO+CCR7+), EM (CD45RO+CCR7−CD27−) and TM (CD45RO+CCR7-CD27+). Gates were defined using fluorescence minus one (FMO) controls and uninfected controls.

### Statistical analysis

All statistical analyses were performed using GraphPad Prism v7.0. To determine differences among more than 2 unpaired groups of different size, a non-parametric Kruskal–Wallis test with Dunn’s multiple comparison post-test was performed. Correlations were plotted as Spearman correlations. *p* values < 0.05 were considered statistically significant. *p < 0.05; **p < 0.01; ***p < 0.001; ****p <0.0001.

## Supplementary information

**Additional file 1: Table S1.** List of sequences and Genbank accession numbers used in this study. **Table S2.** Primers used to amplify and clone C-HIV Envs into pSVIII-Env. **Table S3.** Coreceptor usage of C-HIV Envs. **Figure S1.** Sequence alignments of V3 regions from each study participant. **Figure S2.** AMD3100 inhibition of CXCR4 virus entry. **Figure S3.** Gating strategy used for identifying infected CD4^+^ T cell subsets as a percentage of total infected cells. **Figure S4.** Gating strategy used for identifying CD4^+^ T cell subset infection level. **Figure S5.** Contribution of each CD4^+^ T cell subset to the pool of productively infected CD4^+^ T cells. **Figure S6.** Increased CCR5 expression in more differentiated CD4^+^ T cell subsets.

## Data Availability

Sequences of *env* clones that were used in this study can be found in the GenBank nucleotide repository under the accession numbers shown in Additional file 1: Table S1.

## Abbreviations

HIV-1: Human immunodeficiency virus type 1
C-HIV: Subtype C HIV-1
TSCM: T stem cell memory
CM: Central memory
TM: Transitional memory
EM: Effector memory
TD: Terminally differentiated
Env: Envelope
CAPRISA: Centre for the AIDS Programme of Research in South Africa
GFP: Green fluorescent protein
PNLGs: Potential N-linked glycosylation sites
CRUSH: CoReceptor USage prediction for HIV-1
T0: Enrolment/acute infection
T1: 1-year post-infection
T3: 3-years post-infection
SGA: Single genome amplification
R5: CCR5
X4: CXCR4
