# Supplementary material for: Longitudinal analysis of subtype C envelope tropism for memory CD4+ T cell subsets over the first 3 years of untreated HIV-1 infection
Source: Retrovirology. 2020 Aug 6;17:24. doi: 10.1186/s12977-020-00532-2 (PMC7409430; doi:10.1186/s12977-020-00532-2)
Supplement: Supplementary file 1 — Additional file 1: Table S1. List of sequences and Genbank accession numbers used in this study. Table S2. Primers used to amplify and clone C-HIV Envs into pSVIII-Env. Table S3. Coreceptor usage of C-HIV Envs. Figure S1. Sequence alignments of V3 regions from each study participant. Figure S2. AMD3100 inhibition of CXCR4 virus entry. Figure S3. Gating strategy used for identifying infected CD4+ T cell subsets as a percentage of total infected cells. Figure S4. Gating strategy used for identifying CD4+ T cell subset infection level. Figure S5. Contribution of each CD4+ T cell subset to the pool of productively infected CD4+ T cells. Figure S6. Increased CCR5 expression in more differentiated CD4+ T cell subsets. [file 12977_2020_532_MOESM1_ESM.docx]

**Gartner et al.**

**Supplementary Material**

**Additional file 1: Table S1: List of sequences and Genbank accession numbers used in this study.**

**Additional file 1: Table S2: Primers used to amplify and clone C-HIV Envs into pSVIII-Env**

**Additional file 1: Table S3: Coreceptor usage of C-HIV Envs**

**Additional file 1: Figure S1: Sequence alignments of V3 regions from each study participant.** V3 amino acid sequences were curated and aligned for a) CAP88, b) CAP177, c) CAP228, d) CAP255 and e) CAP257. Dots represent amino acids that are identical to the top sequence in the alignment (T0 sequence).

**Additional file 1: Figure S2:** **AMD3100 inhibition of CXCR4 virus entry.** NP2/CD4/CXCR4 cells were treated with 5uM of AMD3100 before infection with equivalent amounts of Env-pseudotyped luciferase reporter virus in triplicate. Each data point represents the average percentage of viral entry compared to no inhibitor controls for an individual experiment with the bars representing median ± interquartile range (n=3).

**Additional file 1: Figure S3: Gating strategy used for identifying infected CD4^+^ T cell subsets as a percentage of total infected cells.** GFP reporter virus pseudotyped with CAP228.4.25.13-10A Env is shown in this example. Cells were first gated using a forward (FSC-A) and side (SSC-A) scatter gate and then on single cells using FCS-A and FCS-H. Viable T cells were then gated using a viability dye (Viability dye eF506) and CD3 APC-Cy7. The CD4^+^ T cells were detected for infectivity by gating on double positive CD3 APC-Cy7 and GFP. The GFP+CD3+ T cells were then separated into the following CD4^+^ T cell subsets; naïve-like, terminally differentiated (TD, CD3+CD45RO-CCR7-), central memory (CM, CD3+CD45RO+CCR7+), and transitional memory (TM) + effector memory (EM) cells by using CD45RO eF450 and CCR7 AF647. TM + EM gated cells were further separated into TM (CD3+CD45RO+CCR7-CD27+) and EM (CD3+CD45RO+CCR7-CD27-) subsets by using CD27 PE-Cy7. TSCM cells (CD3+CD45RO-CCR7+CD95+CD122+) were separated from naïve cells (CD3+CCR7-CD45RO-) in the naïve-like population using CD122 PerCP-eF710 and CD95 PE-CF594.

**Additional file 1: Figure S4: Gating strategy used for identifying CD4^+^ T cell subset infection level.** GFP reporter virus pseudotyped with CAP228.4.25.13-10A Env is shown in this example. Cells were first gated using a forward (FSC-A) and side (SSC-A) scatter gate and then on single cells using FCS-A and FCS-H. Viable T cells were gated using a viability dye (Viability dye eF506) and CD3 APC-Cy7. CD3+ T cells were separated into the following CD4^+^ T cell subsets; naïve (CD3+CD45RO-CCR7+), terminally differentiated (TD, CD3+CD45RO-CCR7-), central memory (CM, CD3+CD45RO+CCR7+), and transitional memory (TM) + effector memory (EM) cells by using CD45RO eF450 and CCR7 AF647. TM + EM gated cells were further separated into TM (CD3+CD45RO+CCR7-CD27+) and EM CD3+CD45RO+CCR7-CD27-) subsets by using CD27 PE-Cy7. TSCM cells (CD3+CD45RO-CCR7+CD95+CD122+) were separated from naïve cells (CD3+CCR7-CD45RO-) in the naïve-like population using CD122 PerCP-eF710 and CD95 PE-CF594. Infection of each subset was then detected through gating on GFP+ cells.

**Additional file 1: Figure S5:** **Contribution of each CD4^+^ T cell subset to the pool of productively infected CD4^+^ T cells.** Each data point represents each subset infected (as a percentage of the total CD4^+^ T cells infected) averaged across four seronegative donors. Each graph represents a different CD4^+^ T cell subset; a) naïve, b) T stem cell memory (TSCM), c) central memory (CM), d) transitional memory (TM), e) effector memory (EM), and f) terminally differentiated (TD). Each data set is stratified by the Env donor and time point the Env was obtained. Black lines represent median values. Statistical comparisons were not made given the small number of Envs per time point and donor.

**Additional file 1: Figure S6: Increased CCR5 expression in more differentiated CD4^+^ T cell subsets.** CCR5 expression on the indicated CD4^+^ T cell subset was determined by mean fluorescence intensity (MFI) after CD4^+^ T cell isolation and before infection. Each data point represents the CCR5 MFI from a single HIV-seronegative donor with lines representing the median.

| **Time Point** | **Sequence ID** | **Accession Number** | **Reference** |
| --- | --- | --- | --- |
| Acute | CAP177.1.00.G | MK205532.1 | (1) |
| 1 Year | CAP177.3.16.12.1.10D | MK205561.1 | (1) |
|  | CAP177.3.16.12.11.3A | MK205566.1 | (1) |
|  | CAP177.3.16.12.47.6F | MK205571.1 | (1) |
|  | CAP177.3.16.28.5C | MT260176 |  |
| 3 Years | CAP177.4.25.1.11E | MT260177 |  |
|  | CAP177.4.25.28.7A | MT260178 |  |
|  | CAP177.4.25.30.10G | MT260179 |  |
|  | CAP177.4.25.53.1F | MT260180 |  |
| Acute | CAP88.2.00.17.5a | MK206069.1 | (1) |
| 1 Year | CAP88.12.2.2F | MK205512.1 | (1) |
|  | CAP88.12.17.7F | MK205575.1 | (1) |
|  | CAP88.12.21.10D | MK205514.1 | (1) |
|  | CAP88.12.23.7A | MK205564.1 | (1) |
|  | CAP88.12.45.10B | MK205531.1 | (1) |
|  | CAP88.12.46.4B | MK205520.1 | (1) |
| 3 Years | CAP88.4.25.H10 | MK205649.1 | (1) |
|  | CAP88.4.25.G3-3 | MK205653.1 | (1) |
|  | CAP88.4.25.D5-4 | MK205648.1 | (1) |
|  | CAP88.4.25.A3-4 | MK205654.1 | (1) |
| Acute | CAP228.2.00.18 | EF203969.1 | (2) |
|  | CAP228.2.00.51 | EF203968.1 | (2) |
| 1 Year | CAP228.3.15.4a | MK206188.1 | (1) |
|  | CAP228.3.15.8d | MK206191.1 | (1) |
|  | CAP228.3.15.13c | MK206193.1 | (1) |
|  | CAP228.3.15.2-2e | MT260181 |  |
| 3 Years | CAP228.4.25.13-10A | MT260182 |  |
|  | CAP228.4.25.7-9A | MT260183 |  |
|  | CAP228.4.25.5-5d | MK205477.1 | (1) |
|  | CAP228.4.25.4-3F | MT260184 |  |
| Acute | CAP255.2.00.16 | EF203982.1 | (2) |
| 1 Year | CAP255.3.16.10.Q | MK205974.1 | (1) |
|  | CAP255.3.16.13.T | MK205971.1 | (1) |
|  | CAP255.3.16.3.O | MK205977.1 | (1) |
|  | CAP255.3.16.2.N | MK205966.1 | (1) |
| 3 Years | CAP255.4.25.34-7D | MK206047.1 | (1) |
|  | CAP255.4.25.23-3D | MK206037.1 | (1) |
|  | CAP255.4.25.95-2C | MK206041.1 | (1) |
|  | CAP255.4.25.9-11D | MK206042.1 | (1) |
| Acute | CAP257.2.00.L | MK206079.1 | (1) |
| 1 Year | CAP257.3.16.A | MK206103.1 | (1) |
|  | CAP257.3.16.C | MK206104.1 | (1) |
|  | CAP257.3.16.D | MK206105.1 | (1) |
|  | CAP257.3.16.F | MK206106.1 | (1) |
|  | CAP257.3.16.G | MK206107.1 | (1) |
|  | CAP257.3.16.0-1 | MK206110.1 | (1) |
| 3 Years | CAP257.4.25.B6 | MK206120.1 | (1) |
|  | CAP257.4.25.G10 | MK206114.1 | (1) |
|  | CAP257.4.25.H10 | MK206113.1 | (1) |
|  | CAP257.4.25.G9 | MK206118.1 | (1) |

**Additional file 1: Table S1: List of sequences and Genbank accession numbers used in this study.**

**Additional file 1: Table S2: Primers used to amplify and clone C-HIV Envs into pSVIII-Env**

| Forward primers | 5' to 3' |
| --- | --- |
| Kpn-F2 CAPRISA | GGGGTACCTGTGTGGAAAGAAGC |
| Kpn-F2 CAP228 | GGGGTACCTGTGTGGACAGACGC |
| Kpn-F2 CAP255 | GGGGTACCTGTGTGGAGAGAAGC |
| Kpn-F2 CAP228 3.15 2e | GGGGTACCTGTATGGACAGACGC |
| Kpn-F2 CAP255 | GGGGTACCTGTGTGGAGAGAAGC |
| Kpn-F2 CAP255 3.16 10.Q | GGGGTACCTGTGTGGAAAGAAGC |
| Kpn-F2 CAP255 3.16 2.N | GGGGTACCTGTGTGGAGAGACGC |
| Reverse primers | **5' to 3'** |
| Env-Bam CAP88 Rev | GATAAGGATCCGCTCACTAATCGA |
| Env-Bam CAP88 1yr Rev | GCTAAGGATCCGCTCACTAATCGA |
| Env-Bam CAP88 1yr 3yr Rev | GACAAGGATCCGCTCACTAATCGA |
| Env-Bam CAP177 Rev | GATAAGGATCCGTTCACTAATCGA |
| Env-Bam CAP228 Rev | GCTAAGGATCCGTTCACTAATCGA |
| Env-Bam CAP228 3.15 2e Rev | GCTAAGGATCCGTTCGCTAATCGA |
| Env-Bam CAP255 Rev | GCTAAGGATCCGCTCACTAATCGC |
| Env-Bam CAP257 early Rev | GACAAGGATCCGTTCACTAATCGA |
| Env-Bam CAP257 3yr Rev | GACAAGGATCCGCTCACTAATCGA |
| Env-Bam CAP257 4.25 G10 Rev | GACAAGGATCCGTTCACTAATCGC |

*Acc65I* site underlined in forward primers and *BamHI* site underlined in reverse primers.

**Additional file 1: Table S3: Coreceptor usage of C-HIV Envs**

| **Participant** | **Time point** | **Virus ID** | **Coreceptor Prediction** | **Tropism** | **CCR5 Usage** | **CXCR4 Usage** |
| --- | --- | --- | --- | --- | --- | --- |
| **CAP177** | Enrolment | CAP177.1.00.G | R5 | R5 | +++ | - |
|  | 1 Year | CAP177.3.16.12.1.10D | R5 | R5 | +++ | - |
|  |  | CAP177.3.16.12.11.3A | R5 | R5 | +++ | - |
|  |  | CAP177.3.16.12.47.6F | R5 | R5 | +++ | - |
|  |  | CAP177.3.16.28.5C | R5 | * | - | - |
|  | 3 Years | CAP177.4.25.1.11E | R5 | R5 | +++ | - |
|  |  | CAP177.4.25.28.7A | R5 | R5 | +++ | - |
|  |  | CAP177.4.25.30.10G | R5 | R5 | +++ | - |
|  |  | CAP177.4.25.53.1F | R5 | R5 | +++ | - |
| **CAP88** | Enrolment | CAP88.2.00.17.5a | R5 | R5 | +++ | - |
|  | 1 Year | CAP88.12.2.2F | R5 | R5 | +++ | - |
|  |  | CAP88.12.17.7F | R5 | R5/X4 | +++ | ++ |
|  |  | CAP88.12.21.10D | R5 | R5 | ++ | - |
|  |  | CAP88.12.23.7A | R5 | R5/X4 | ++ | + |
|  |  | CAP88.12.45.10B | R5 | R5 | +++ | - |
|  |  | CAP88.12.46.4B | R5 | R5/X4 | +++ | + |
|  | 3 Years | CAP88.4.25.H10 | R5 | R5 | +++ | - |
|  |  | CAP88.4.25.G3-3 | R5 | R5 | +++ | - |
|  |  | CAP88.4.25.D5-4 | R5 | R5 | +++ | - |
|  |  | CAP88.4.25.A3-4 | R5 | R5 | +++ | - |
| **CAP228** | Enrolment | CAP228.2.00.18 | R5 | R5 | +++ | - |
|  |  | CAP228.2.00.51 | R5 | R5 | +++ | - |
|  | 1 Year | CAP228.3.15.4a | R5 | R5 | +++ | - |
|  |  | CAP228.3.15.8d | R5 | R5 | ++ | - |
|  |  | CAP228.3.15.13c | R5 | R5 | +++ | - |
|  |  | CAP228.3.15.2-2e | R5 | R5 | +++ | - |
|  | 3 Years | CAP228.4.25.13-10A | R5 | R5 | +++ | - |
|  |  | CAP228.4.25.7-9A | R5 | * | - | - |
|  |  | CAP228.4.25.5-5d | R5 | R5 | +++ | - |
|  |  | CAP228.4.25.4-3F | R5 | R5 | +++ | - |

**Additional file 1: Table S3 (continued): Coreceptor usage of C-HIV Envs.**

R5: CCR5, X4: CXCR4

| **Participant** | **Time point** | **Virus ID** | **Coreceptor Prediction** | **Tropism** | **CCR5 Usage** | **CXCR4 Usage** |
| --- | --- | --- | --- | --- | --- | --- |
| **CAP255** | Enrolment | CAP255.2.00.16 | R5 | R5 | ++ | - |
|  | 1 Year | CAP255.3.16.10.Q | R5 | R5 | ++ | - |
|  |  | CAP255.3.16.13.T | R5 | R5 | ++ | - |
|  |  | CAP255.3.16.3.O | R5 | R5 | +++ | - |
|  |  | CAP255.3.16.2.N | R5 | * | - | - |
|  | 3 Years | CAP255.4.25.34-7D | R5 | R5 | +++ | - |
|  |  | CAP255.4.25.23-3D | R5 | R5 | +++ | - |
|  |  | CAP255.4.25.95-2C | R5 | R5 | ++ | - |
|  |  | CAP255.4.25.9-11D | R5 | R5 | +++ | - |
| **CAP257** | Enrolment | CAP257.2.00.L | R5 | R5 | +++ | - |
|  | 1 Year | CAP257.3.16.A | R5 | R5 | ++ | - |
|  |  | CAP257.3.16.C | R5 | R5 | +++ | - |
|  |  | CAP257.3.16.D | R5 | * | - | - |
|  |  | CAP257.3.16.F | R5 | R5 | +++ | - |
|  |  | CAP257.3.16.G | R5 | R5 | +++ | - |
|  |  | CAP257.3.16.0-1 | R5 | R5 | +++ | - |
|  | 3 Years | CAP257.4.25.B6 | R5 | R5 | +++ | - |
|  |  | CAP257.4.25.G10 | R5 | R5 | +++ | - |
|  |  | CAP257.4.25.H10 | R5 | R5 | +++ | - |
|  |  | CAP257.4.25.G9 | R5 | * | - | - |
|  |  |  |  |  |  |  |

The level of virus entry was scored at the 1/2 dilution as – (<10,000 relative luminescence units (RLU)), + (10,000-100,000 RLU), ++ (100,000 – 1,000,000 RLU), or +++ (>1,000,000 RLU).

* Infectivity was too low to appropriately assess virus phenotype.


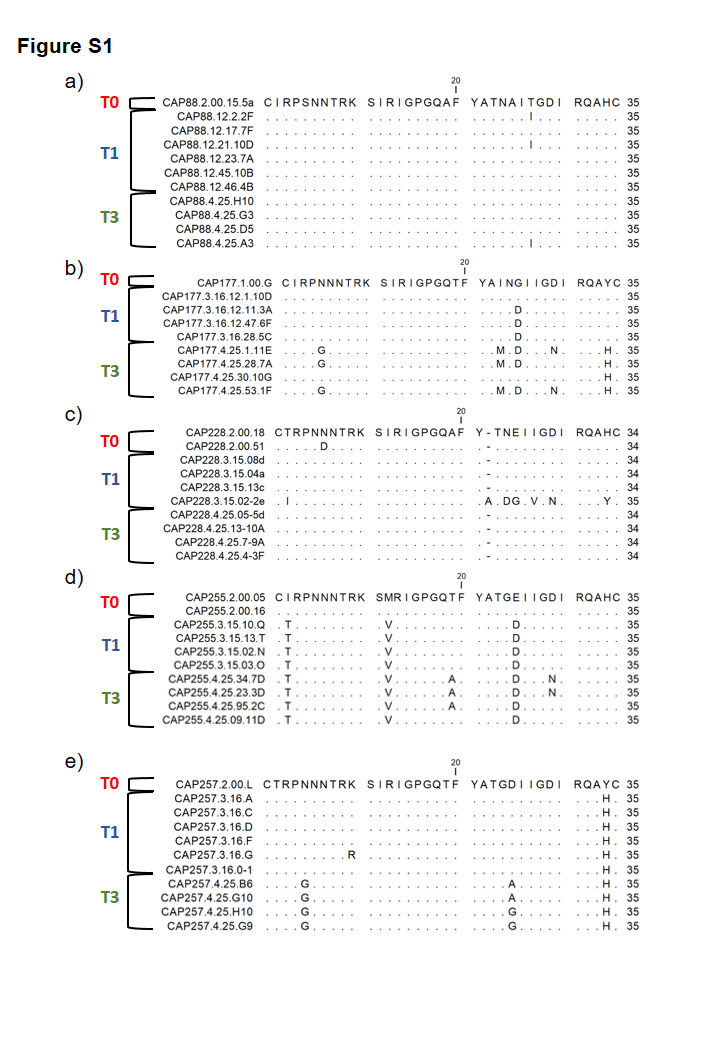


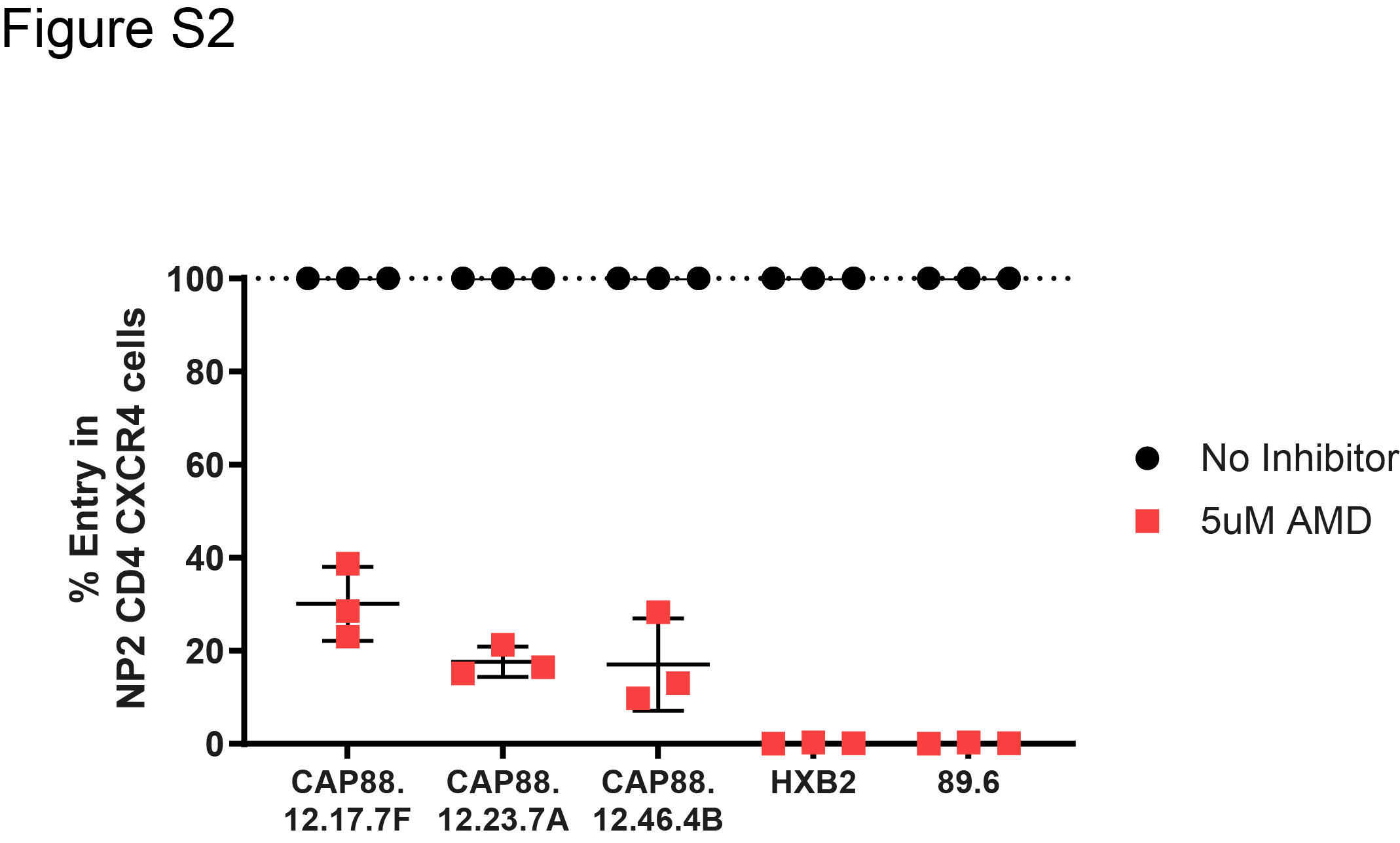


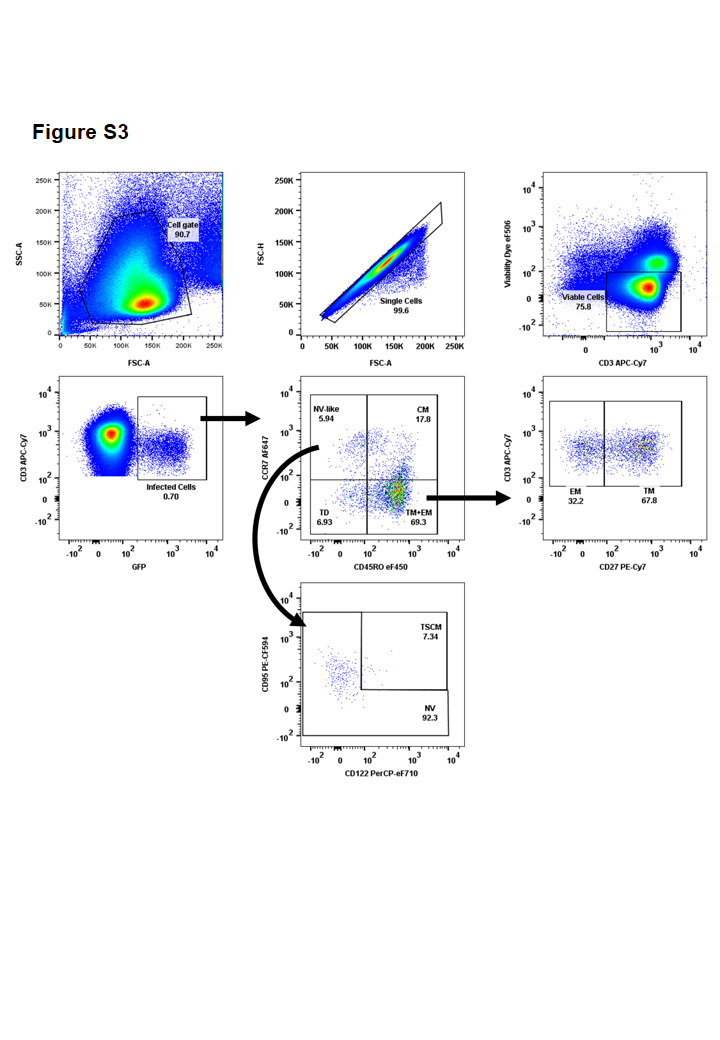


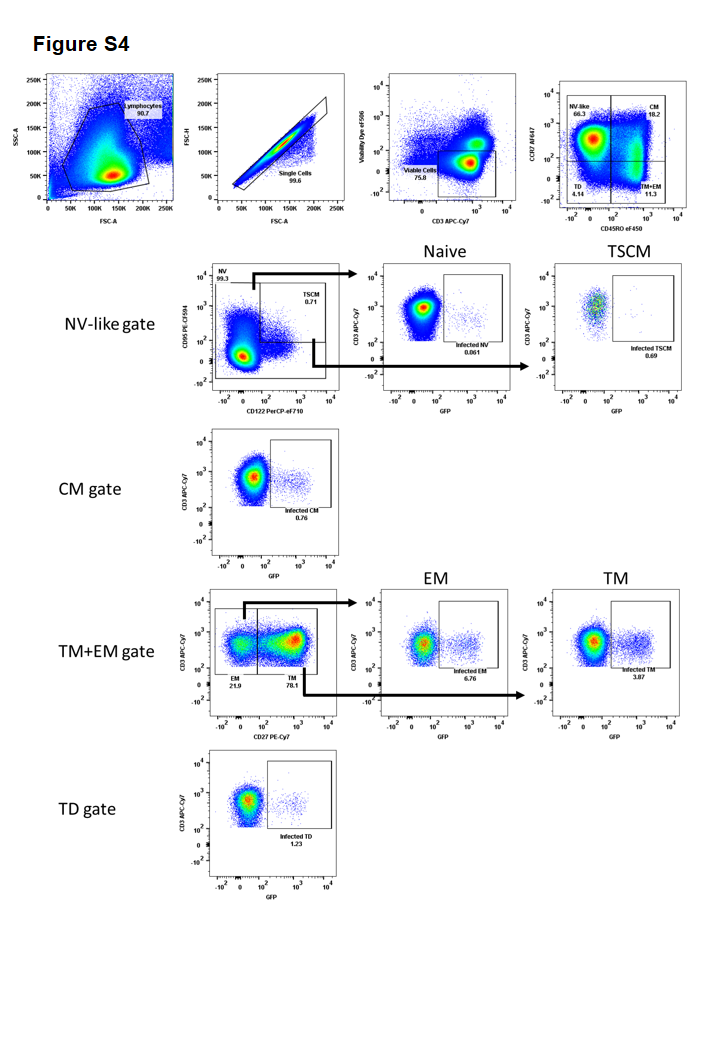


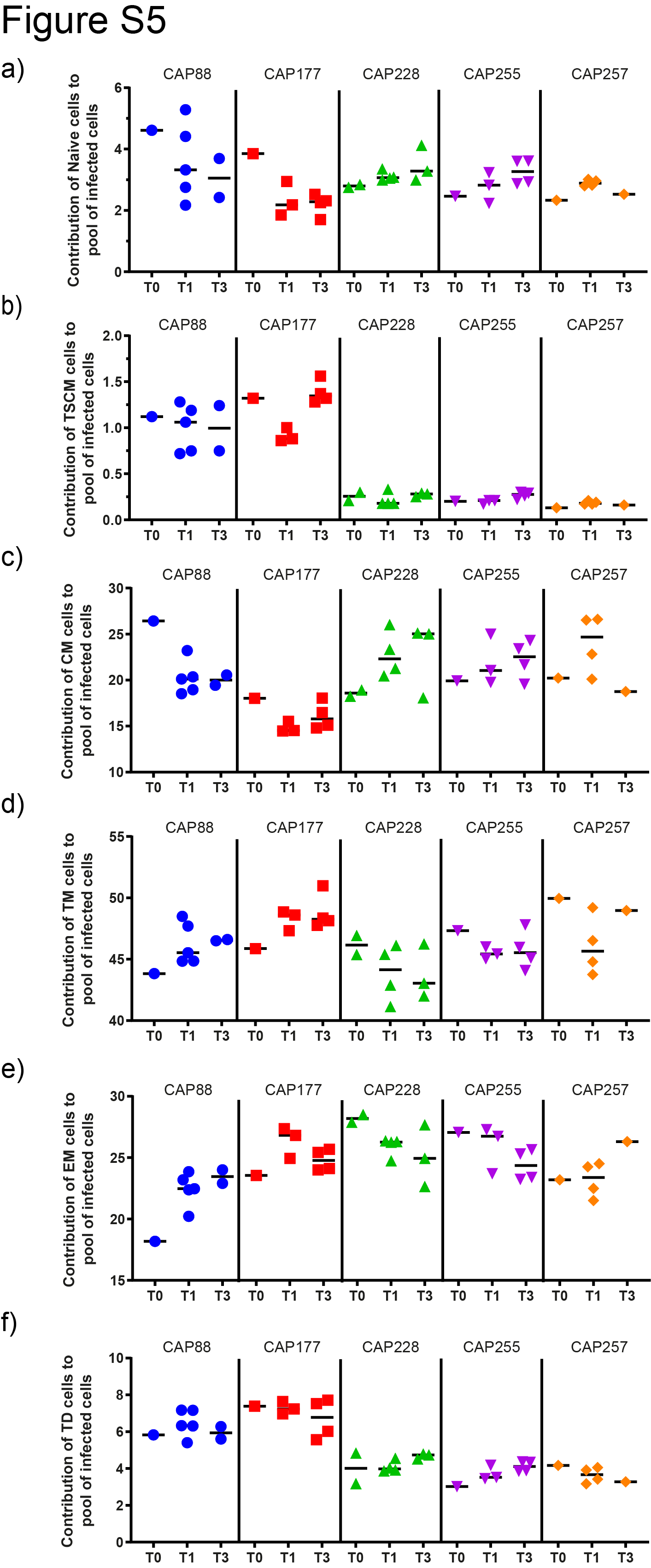


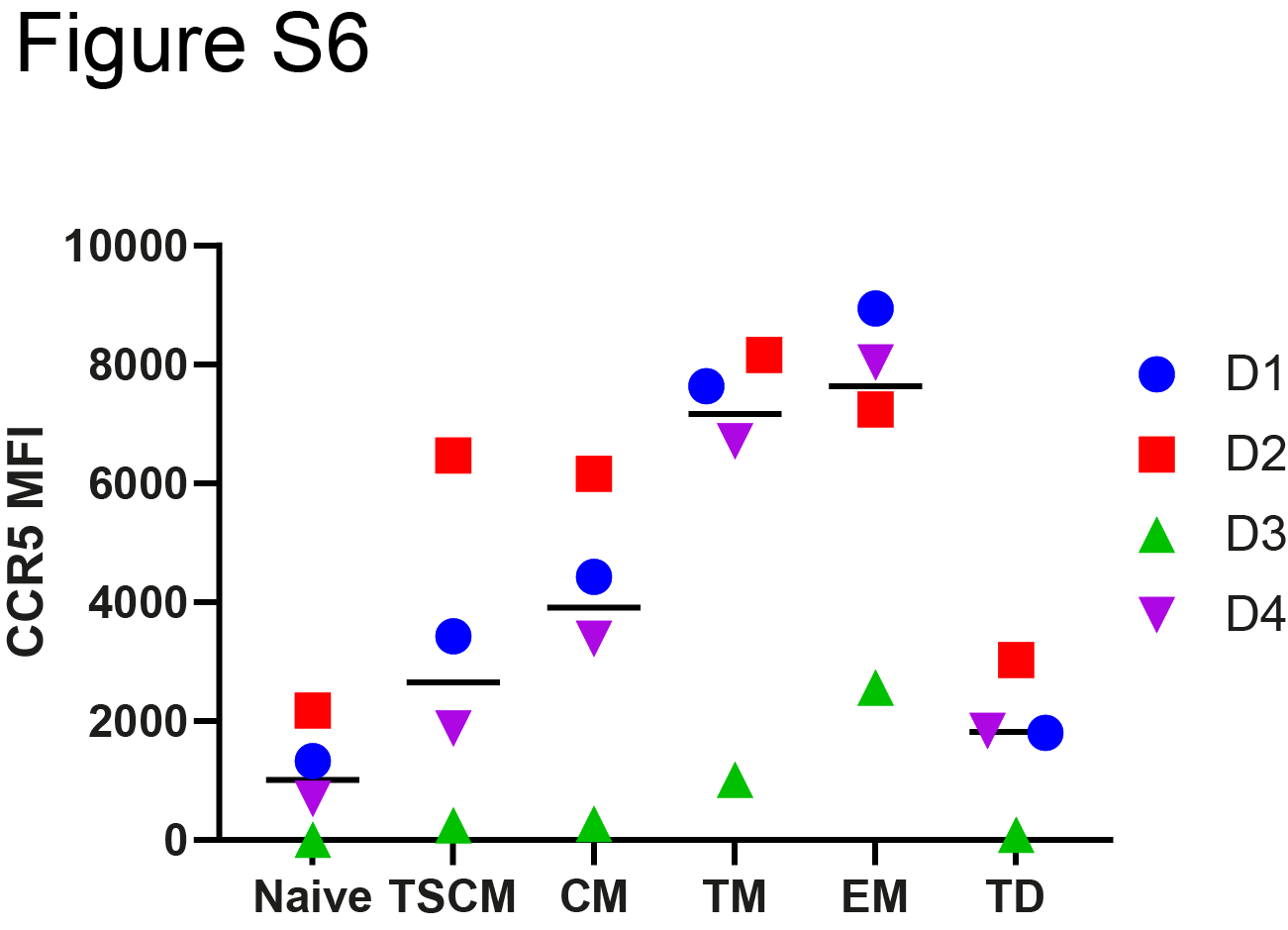


**References**

1. Mabvakure BM, Scheepers C, Garrett N, Abdool Karim S, Williamson C, Morris L, et al. Positive Selection at Key Residues in the HIV Envelope Distinguishes Broad and Strain-Specific Plasma Neutralizing Antibodies. *J Virol.* 2019;**93**(6): e01685-18.

2. Gray ES, Moore PL, Choge IA, Decker JM, Bibollet-Ruche F, Li H, et al. Neutralizing antibody responses in acute human immunodeficiency virus type 1 subtype C infection. *J Virol.* 2007;**81**(12):6187-96.
